# Elucidating the Pivotal Neuroimmunomodulation of Stem Cells in Spinal Cord Injury Repair

**DOI:** 10.1155/2021/9230866

**Published:** 2021-07-23

**Authors:** Seidu A. Richard, Marian Sackey

**Affiliations:** ^1^Department of Medicine, Princefield University, P.O. Box MA128, Ho, Ghana; ^2^Department of Pharmacy, Ho Teaching Hospital, P.O. Box MA-374, Ho, Ghana

## Abstract

Spinal cord injury (SCI) is a distressing incident with abrupt onset of the motor as well as sensory dysfunction, and most often, the injury occurs as result of high-energy or velocity accidents as well as contact sports and falls in the elderly. The key challenges associated with nerve repair are the lack of self-repair as well as neurotrophic factors and primary and secondary neuronal apoptosis, as well as factors that prevent the regeneration of axons locally. Neurons that survive the initial traumatic damage may be lost due to pathogenic activities like neuroinflammation and apoptosis. Implanted stem cells are capable of differentiating into neural cells that replace injured cells as well as offer local neurotrophic factors that aid neuroprotection, immunomodulation, axonal sprouting, axonal regeneration, and remyelination. At the microenvironment of SCI, stem cells are capable of producing growth factors like brain-derived neurotrophic factor and nerve growth factor which triggers neuronal survival as well as axonal regrowth. Although stem cells have proven to be of therapeutic value in SCI, the major disadvantage of some of the cell types is the risk for tumorigenicity due to the contamination of undifferentiated cells prior to transplantation. Local administration of stem cells via either direct cellular injection into the spinal cord parenchyma or intrathecal administration into the subarachnoid space is currently the best transplantation modality for stem cells during SCI.

## 1. Introduction

Spinal cord injury (SCI) is a distressing incident with abrupt onset of the motor as well as sensory dysfunction [1, 2]. SCI most often occurs as a result of high-energy or velocity accidents as well as contact sports and falls in the elderly [2, 3]. Initially, SCIs were mostly seen in young patients but the trend is currently increasing in elderly patients resulting in cervical canal stenosis [3]. SCI is often associated with personal losses by the patients and their families as well as substantial societal cost [1, 2]. The injury usually results in damage to autonomic neurons at and below the cord resulting in bowel, bladder, and sexual dysfunctions [1, 2].

Human SCI remains a serious challenge with currently no successful treatments [1, 4]. Nevertheless, surgical intervention and subsequent rehabilitation are the only alternatives for SCI treatment. Furthermore, although methylprednisolone is usually given to patients at the acute stage of injury, a consensus of its usage is still a matter of debate in terms of both safety and effectiveness [1, 5, 6]. Stem cell transplantation may provide an effective treatment for SCI due to the self-renewing and multipotential nature of these cells [7]. Thus, analytical hypotheses to consider in translating stem cell therapies for SCI comprise injury severity; cell type; spinal level such as cervical, thoracic, and lumber levels; cell delivery system, and epicenter and/or perilesional injections.

This review therefore explores the key roles of stem cell transplantation for spinal cord injury repair with a focus on the parameters above with the key focus on the influence that the microenvironment has after stem cell transplantation in SCI. The “boolean logic” was utilized to search for the article on the subject matter. Most of the articles were indexed in PubMed with strict inclusion criteria being the type of cells and the mode of delivery as well as the effectiveness or success after transplantation. The search terms were functional anatomy of the spine, spinal dynamics, and stem cell transplantation and/or SCI in animal models and humans.

## 2. Functional Anatomy of the Spine

The human spine is a complex column with a combination of substantial structural support and restrictive motions throughout its 24 articulating vertebrae [8, 9]. The cervical spine is very flexible, strong, and mobile in all directions and thus functions as the sensory unit as well support of the head. It permits the sensory structures of the vision, hearing, and smell to move freely in the sagittal plane and also articulate with the environment in the horizontal plane [9, 10]. The cervical spine is long and slender and therefore susceptible to either minor or major injuries [9]. The cervical spine is often divided into three zones which differ both in structure and in function. These divisions include the suboccipital zone (C1 vertebra), a transitional zone (C2 vertebra), and the typical zone (C–7 vertebrae) [9, 11].

The thoracic vertebrae have vertebral bodies that are joined by intervertebral discs as well as longitudinal ligaments and posterior elements that are joined by zygapophysial joints just like cervical and lumbar vertebrae [9]. In all, there are 12 thoracic vertebrae in the human body [8, 9]. The nerves control motor as well as sensory signals in the upper back, chest, and abdomen [9]. Nevertheless, exceptions of the thoracic vertebra arrangement occur at T1 and at T11 and T12, where the head of the rib completely articulates with the like-numbered vertebrae [9]. The essential role of the lumbar spine is to support the thorax as well as the upper limbs. The lumber spine aids load carrying and transmits the weight of loads to the pelvis and lower limbs [9, 12].

Also, the lumbar spine supports a very little range of movement between the thorax and pelvis [13]. In all, there are 5 lumber vertebrae in the human body [12]. The human sacrum is a huge triangular bone comprising of five separate vertebra that fuse along with the intervening intervertebral discs [13]. The sacrum fuses with four bones: the last lumbar vertebra upwards through a disc space as well as the facet joint complex, the coccyx downwards with a ligamentous attachment and seldomly a bone union, and on either side with the ilium via the sacroiliac joint [13].

## 3. Spinal Dynamics and Injury

The spinal cord is a modeled cylinder comprising of diverse anisotropic elastic dense tissue, with a fibrous surface lining (meninges), suspended in fluid, tethered via small ligaments, and having an intermittent motion as well as tissue waves related to cardiac pulsation and respiration [14–16]. The tethering in the spinal cord is via the denticulate ligaments between the pia mater and dura mater [17, 18]. Cardiac pulsation often triggers longitudinal pulsatile motions in the spinal cord and in connection with cerebrospinal fluid (CSF) flow; the spinal cord also experiences small fluctuations in the area [14, 19–21]. Spinal cord motion comprises of the direction, magnitude of total displacement, and velocity of motion [14, 22, 23]. The CSF flow is often correlating with cycles of cranial as well as caudal cord motion in a normal spinal cord [14, 24–26]. Normally, nerve roots do not come under tension during physiological motion in an intact spinal cord and thus do not exhibit any pain symptoms until during an injury [14]. Therefore, spinal cord motion may be reduced at the injury site due to subarachnoid scarring [14].

SCI characteristically has an injury epicenter where there is emergent tissue necrosis as well as cavity formation, axonal demyelination, glial stimulation, axotomy and scarring, and analogous endogenous repair activities such as neoangiogenesis and axonal sprouting [14, 27]. Cord swelling, inflammation, and tissue softening with areas of necrosis which ultimately become a cavity are the pathological processes during the acute and subacute periods after a severe SCI [14, 28, 29]. Acute SCI often results in vascular changes with loss of neurons, oligodendroglia, and astrocytes [3]. Also, neuroinflammation occurs with resultant invasion of the injury by a variability of inflammatory cells. Inflammatory cascades such as neutrophils, macrophages/microglia, and T-cells, as well as humoral components like cytokines, interleukins, interferons, and prostaglandins, are often triggered during SCI [3]. Apart from the triggering of inflammatory cascades, the acute phase also involves hemorrhage, ischemia, excitotoxicity, and oxidative stress resulting in secondary cell death and degeneration of more tissue [3, 30].

The acute phase is associated to Wallerian degeneration of ascending and descending tracts with gradual formation of cavities in the cord, and the formation of the glial scar decreases significantly the growth capabilities of axons across the injury [31–33]. The blockade of nerve conduction results in paralysis as well as temporary loss of neural functions by spinal shock [3, 34, 35]. The associated oxidative stress results in the reduction of glutamate transport in astrocytes, thereby stimulating excitotoxicity because of augmented extracellular glutamate [3, 36]. The resultant ischemia triggers necrotic cell death in the epicenter of the injury. This process is usually the mechanism via which the induction of destructive signaling cascade occurs and expands to cause tissue damage [3, 37–39]. In response to edema, several vasoactive factors such as thromboxane, leukotrienes, platelet aggregation factors, serotonin and endogenous opioids are released [3, 40, 41]. This mechanism results in hypoperfusion, hypoxia, and hypoglycemia [3, 40, 41].

After ischemia follows a period of reperfusion which results in an increase in free reactive oxygen species (ROS) [3, 42, 43]. The generation and release of ROS are often the mechanisms via which the secondary injury process and the maintenance of a degenerative environment occur [3, 43]. On the other hand, complete loss of a grey matter with some preserved parenchyma, a margin of pia and fibroblastic scar, and an underlying thin rim of preserved gliotic white matter are the pathological cascade at the epicenter of most damaged spinal cords during the chronic period [3, 44–46]. An ideal treatment must be efficient in triggering axon regeneration in the injured central spinal cord and also attenuating scarring. It must also be capable of the generating growth-inhibitory factors at the lesion site as well as stimulating axon growth [32, 47, 48].

## 4. Neural Stem Cells

**Figure 1:**
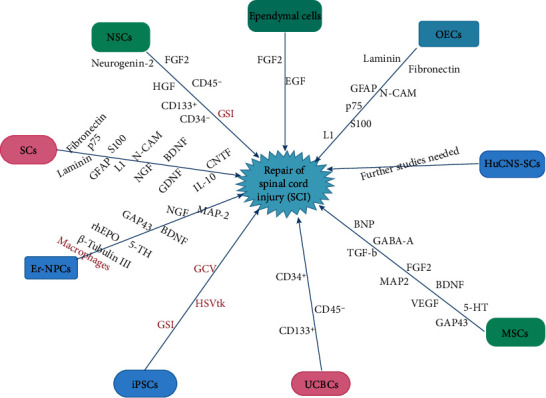
An illustration of the various types of stem cells and the pathways via which they influence the repair of spinal cord injury. Red: inhibitory pathway/tumor inhibition; black: facilitatory pathway; NSCs: neural stem cells; SCs: Schwann cells; OECs: olfactory ensheathing cells; Er-NPCs: erythropoietin-releasing neural precursors cells; UCBCs: umbilical cord blood cells; iPSCs: induced pluripotent stem cells; HuCNS-SCs: human central nervous system stem cells; MSCs: mesenchymal stem cells.

Neural stem cells (NSCs) (Figure 1) have been obtained from various regions of the brain from mice, rats, monkeys, and humans [3, 49]. Fetal NSCs and adult NSCs are the main types of NSCs. Fetal NSCs can be expanded for a long period *in vitro*, while adult NSCs have more partial abilities [49, 50]. Nevertheless, both cell types have an ineffective differentiation potential into neurons after numerous *in vitro* routes when they are transplanted into *in vivo* models [51, 52]. Several studies have demonstrated that adult NSCs are located in the spinal cord and usually around the central canal with narrow extension to the ventricular system stretching across the length of the spinal cord [53–56]. Analogous clones were capable of proliferating from both medial and lateral parts of the spinal cord [57, 58]. Nevertheless, minor multipotencies with few passages were observed during the later parts of the coning process [57, 58].

NSC proliferation in the spinal cord differs from that of the NSCs from the forebrain, where neurogenesis was observed to be sustained throughout the organism life [58]. NSCs from the spinal cord also require a distinctive mitogen *in vitro*, fibroblast growth factor-2 (FGF2) (Table 1) instead of epidermal growth factor (EGF) utilized for NSCs from the brain [55]. Human NSCs were cultured as neurospheres survived, migrated, and secreted differentiation markers for neurons and oligodendrocytes (Table 1) after long-term transplantation in SCI [59, 60]. Transplantation embryonic NSCs in aged mice were capable of improving functional recovery after SCI via the reformation of the cord microenvironment by stimulating the local secretion of growth factors, particularly the hepatocyte growth factor (HGF) (Table 1) [61]. NSCs have demonstrated to be capable of secreting CD133^+^/CD 34^−^/CD45^−^ [62] (Table 1). *In vitro* studies have shown that EGF and FGF2 are fundamental factors in the cell culture conditions that sustained cell division with NSC [63–66].

Perrin et al. transplanted lentiviral-transduced human fetal neural progenitor cells (NPCs) capable of secreting neurogenin-2 (Table 1) into adult rats and observed that functional recovery correlated with partial restoration of serotonin fiber density caudal to the lesion [67]. Studies has shown that transplanted human NPCs obtained from fetal CNS tissues stimulated regeneration of the host corticospinal tract in the spinal cord with motor functional improvement compared to NPCs with brain characteristics [68–71]. Nevertheless, the major disadvantage with NPCs is the risk for tumorigenicity due to the contamination of undifferentiated cells prior to transplantation [72, 73]. Thus, to prevent tumorigenicity, contaminated cells were eliminated using the *γ*-secretase inhibitor (GSI), which inhibits Notch signaling (Table 1) [72]. The status of undifferentiated NPCs is regulated via the Notch signaling, and the blockade of this signaling triggers further maturation as well as neuronal differentiation of NPCs [72].

## 5. Transplant Cells from Neural Origin

Schwann cells (SCs), Olfactory ensheathing cells (OECs), and ependymal cells are the main transplant cells from neural origin [3, 74–76]. SCs and OECs have a number of morphological as well as molecular markers but have distinctive embryonic origins [75, 76]. SCs are derived from the neural crest while OECs originate from the olfactory placode [3, 74]. It is noteworthy that both cell categories secrete p75, GFAP, S100, and cell adhesion molecules like L1 and N-CAM (Table 1) [3, 74]. Furthermore, both cell categories secrete extracellular molecules like fibronectin and laminin [74].

SCs (Figure 1) are the ancillary glial cells of the peripheral nervous system (PNS) which stimulates the formation of myelin sheaths around peripheral axons as well as associated with intimate axonal glial intercommunications that grant axonal maintenance and impulse conduction [3, 77]. SCs often differentiate as well as proliferate and express distinctive neurotrophic factors which offer natural assistance for axonal regeneration after peripheral nerve injury [3]. Neurotrophic factors (Table 1) such as the nerve growth factor (NGF), brain-derived neurotrophic factor (BDNF), glial cell-derived neurotrophic factor (GDNF), and ciliary neurotrophic factor (CNTF) are often expressed by transplanted SCs [78]. These factors are capable of remyelinating injured axons, chaperon-regenerating axons, and accelerate the invasion of host SCs into the injured spinal cord section [79–81].

Some studies have shown that SC transplantation alone resulted in enhanced recovery of locomotory function in rodents while others demonstrated contrary results [82–85]. Furthermore, other studies showed very little recovery when SCs were simultaneous with methylprednisolone, neurotrophins, IL-10 (Table 1), and OECs at the cord stumps [86–88]. In view of these conflicting studies, more studies are warranted in this direction to determine the actual therapeutic roles of SCs in SCI.

OECs (Figure 1) are a distinct category of the glia situated in the olfactory system [89, 90]. In the olfactory system, the renewal of sensory neurons occurs constantly throughout life [89, 90]. OECs are capable of migrating within the central nervous system (CNS) and coexist in an astrocyte-rich locations [90, 91]. Studies have shown that axons of the new neurons are sheathed by OECs that chaperon and assist in their elongation as they cross from the PNS of the olfactory mucosa to the CNS of the olfactory bulb, where axons make new synaptic connections with other neurons [92–94]. OECs also possess neuroprotective abilities such as the expressing of trophic factors, decreasing of astroglial reactivity, and intercommunicating with damaged axonal pathways [94, 95].

Transplanted OECs were capable of remyelinating as well as improving axonal conduction in the demyelinated pathways of the rat SCI models [77, 96]. The effects of transplanted OECs were observable after acute than delayed transplantation. However, current studies demonstrated that chronic transplants are still efficient in the improvement of recovery during SCI [97–99]. Nevertheless, some studies did not find any significant neuroprotective/regenerative role of OECs in SCI [82, 100–102]. In view of these conflicting studies, more studies are warranted in this direction to determine the actual therapeutic roles of OECs in SCI.

Ependymal cells (Figure 1) are the ciliated cells lining the central canal of the spinal cord. These cells propel the CSF as well as form a barrier to the spinal cord parenchyma [7]. Studies have demonstrated that transplanted ependymal cells self-renew in reaction to SCI as well as differentiated into oligodendrocytes and astrocytes [103–105]. Sabelström et al. demonstrated that blockade of ependymal cell proliferation after SCI rigorously impeded glial scar formation and resulted in augmented neuron loss [106]. Moreover, harvested and cultured ependymal cells were efficient in differentiating into astrocytes, oligodendrocytes, and neurons [106].

Kojima and Tator observed an upsurge in proliferation of ependymal cells as well as enhanced functional recovery when they fused growth factors EGF and FGF2 (Table 1) into the central canal after SCI [107]. Thus, they indicated that manipulation of ependymal cell could be a potential alternative to exogenous stem cell transplantation [107]. Other studies have also proven that ependymal cells are the endogenous stem cells in the adult spinal cord and thus form an alternative cell population to earmark for the treatment of SCI [56, 108].

Human central nervous system stem cells (HuCNS-SCs) (Figure 1 and Table 1) have also proven to be successful cells for transplantation after SCI [109]. They can be propagated, cryopreserved, and banked, while maintaining critical biological activity of self-renewal and engraftment, paracrine effects from expressed factors to improve neural plasticity, migration, and trilineage differentiation such as neurons, oligodendroctyes, and astrocytes [109].

## 6. Erythropoietin-Releasing Neural Precursor Cells

Erythropoietin-releasing neural precursors cells (Er-NPCs) (Figure 1) are very promising in the treatment of SCI [31]. These cells were initially referred as postmortem neural precursor cells [31, 110]. Initial studies demonstrated that intravenous infusion of Er-NPCs isolated from the subventricular zone (SVZ) six hours after the donor's death enhanced hind limb functional recovery but the cells were phagocytized by macrophages and the process of recovery stopped [31, 111, 112]. Er-NPCs accumulate at the lesion site and differentiate mainly into cholinergic neuron cells which were capable of protecting the myelin via the reduction of posttraumatic neuroinflammation [113–116].

Studies further demonstrated that Er-NPCs confine to the edges of the injury site where the microenvironment is often absolutely influenced by the prevailing neutralization of reactive inflammation [31, 113, 116]. Studies also showed that the neuroprotective role of transplanted Er-NPCs supports the structural maintenance or neoformation of an auspicious milieu [31]. This is exhibited by the higher maintenance of neuronal markers (Table 1) like *β*-tubulin III and MAP-2 at the lesion site [31]. Also, TH-positive fiber thickness in the ventral segment of the lumbosacral cord of injured Er-NPC-treated mice was much higher than that of saline-treated injured mice [31].

Cerri et al. demonstrated that exogenous administration of rhEPO (Table 1) augmented the protection of TH-positive (Table 1) fibers and sheaths the injured as well as its associated larger descending spinal and ascending cortical evoked potential [117]. This was accompanied by the substantial protection of parenchyma at the lesion site in the spinal cord as well as substantial attenuation of myelin loss in the ventral and medioventral pathways [117]. Furthermore, descending 5-HT and fibers containing catecholamine that distinctly reinnervate the caudal cord were also significantly boosted [116, 117]. Growth-associated protein-43 (GAP43) (Table 1) is a marker of axonal growth cones [31].

Studies have shown that GAP-43 secretion correlated with axon regrowth abilities and its higher secretion in the caudal cord sustained the improved regeneration across the lesion in Er-NPC-transplanted mice [118–120]. Furthermore, studies revealed that Er-NPCs were capable of enhancing the functional recovery as well as supported axon regeneration via the provision of an auspicious environment and the expression of EPO that had influential anti-inflammatory activity which were capable of decreasing the secretion of inflammatory cytokines which resulted in the neutralization of invasion via the inflammatory cells at the injury site [31, 33, 121].

Therefore, Er-NPCs have both anti-inflammatory and neuroprotective actions which lead to spinal tissue sparing as well as a positive microenvironment which allows for axonal regeneration across the injure site [31, 33, 121]. Compared to regular adult NSCs, transplanted Er-NPCs had a higher survival ability in a hostile environment. Studies have shown that Er-NPCs were capable of infiltration with inflammatory cells like macrophages and neutrophils, which influenced secondary degeneration [113, 116]. Also, the local production of growth factors like BDNF and NGF (Table 1) was augmented which resulted in the stimulation of neuronal survival as well as axonal regrowth [116, 122, 123].

## 7. Mesenchymal Stem Cells

Mesenchymal stem cells (MSCs) (Figure 1) compose of cells that are self-renewing and have the ability to differentiate into various mesodermal tissues such as the bone, cartilage, muscle, and fat [4, 124–126]. Several studies have demonstrated that MSCs are capable of differentiating into neurons and glia, therefore favorable trophic agents as well as cell sparing agents [127–129]. They are capable of triggering several neurotrophic factors and cytokines as well as differentiating into several phenotypes [130]. Furthermore, they are capable of influencing inflammation as well as stimulating the generation of reparative growth factors [4, 131, 132]. MSCs were injected directly into the lesion site in most studies. Nevertheless, successful administration of MSCs via intrathecal, intravenous, or even lumbar puncture has also been reported [133–136].

Hofstetter et al. demonstrated that immature astrocytes obtained from bone marrow stromal cells and administered into the injured spinal cord were capable of stimulating the outgrowth of 5HT-positive fibers (Table 1) because they proposed growth-permissive surfaces [137]. Several studies have shown that the positive efficiency of stem cell transplants in the injured CNS was a result of the expression of trophic factors by the engrafted cells [138–140]. In most studies, the MSCs were transplanted during the acute or subacute phase of the injury with good results [138–141]. Nevertheless, in a few studies, the cells were transplanted at the chronic phase after spinal cord contusion with good results [142–144].

Some studies observed that acutely injected bone marrow MSCs stimulated more tissue sparing than delayed injected cells and their influence was observed as cell survival during the first week postinjection [145, 146]. Chopp et al. observed that intramedullary transplantation of MSCs one week after SCI enhanced functional outcome over a five-week period, with a few cells secreting neural markers [128]. Furthermore, studies observed substantial enhancement in neurological outcome at four months after transplantation with augmented GAP43 (Table 1) secretion among reactive astrocytes in the scar boundary as well as SVZs in rat SCI models [128, 147]. MSCs were also capable of augmenting astrocytic survival as well as increased astrocytic BDNF, vascular endothelial growth factor (VEGF), and FGF2 (Table 1) after ischemic injury *in vitro* [4, 148].

Studies have shown that MSCs were able to proliferate as well as migrate into the injured cortex and also secreted markers for both neurons and astrocytes [4, 149, 150]. Transplanted MSCs also secreted both neuronal marker MAP-2 and *γ*-aminobutyric acid A (GABA-A) receptors (Table 1) [4, 151]. Transplanted MSCs were capable of accelerating recovery in SCI via the expression of brain natriuretic peptide (BNP) (Table 1) as well as vasoactive factors which decreased edema and intracranial pressure as well as increased cerebral perfusion [4, 152, 153]. Also, mouse MSCs transplanted into the rat spinal cord moved towards the injury site within four weeks after transplant which was observed *in vivo* using fluorescence tracking with GFP [4, 150]. Furthermore, the migrated cells expressed neuronal or astrocytic markers [4, 150].

MSC-derived Schwann cells and Matrigel, a synthetic scaffold material, were capable of stimulating axonal regeneration as well as functional recovery after total transection of the adult rat spinal cord [4, 154, 155]. MSCs were also capable of triggering the electrophysiological features of neurons which means that MSCs have neuronal replacement potentials [156–158]. Furthermore, some studies observed cosecretion of markers from distinctive neural lineages in the same cells [156, 159, 160]. Transplanted MSCs were also able to trigger high levels of transforming growth factor-*β* (TGF-*β*) (Table 1) which was able to lessen the formation of scar tissue [4, 161, 162].

## 8. Induced Pluripotent Stem Cells

Induced pluripotent stem cells (iPSCs) (Figure 1) show features analogous to those of embryonic stem cells (ESCs) and are capable of generating all three germ layers [1, 73]. Thus, iPSCs are capable of improving ectodermal neural-lineage cells with suitable culture stimulation [1]. Human iPSC-NPCs were able to boost axonal regrowth, angiogenesis, and maintenance of the whole spinal cord [1, 73]. Thus, iPSC-NPCs were able to influence neurological and electrophysiological recovery [1]. Nevertheless, the cellular features differ according to iPSC lines and some of the iPSC-derived NPCs generated tumors after being transplanted into CNS tissues [1]. Relatively, transplanted NPCs differentiated into three neuronal lineages without developing tumors [163]. Thus, insecure and unsteady human iPSC lines are capable of developing tumors after transplantation [163].

Nagoshi et al. observed that when tumorigenic human iPSC-NPCs were preserved with GSI (Table 1) for only one day *in vitro*, they showed neuronal differentiation, decrease in cell proliferation, and downregulation of tumor-related gene secretion [1]. When they grafted them in the SCI model of NOD/SCID mice, the iPSC-NPCs primarily produced mature neurons around the injury site without tumor formation for about 89 days after the grafting [1]. Comparatively, non-GSI-treated NPCs developed tumors as well as regression of motor function [1]. They concluded that pretreatment with GSI was capable of eradicating tumor-stimulating cells in human iPSC-NPCs [1].

Nagoshi et al. transduced the herpes simplex virus type I thymidine kinase (HSVtk) gene into tumorigenic human iPSC-NPCs and observed that HSVtk phosphorylates its prodrug ganciclovir (GCV) (Table 1) resulting in the generation of cytotoxic GCV phosphate which eliminated immature and/or proliferating tumor cells whilst sparing postmitotic mature neural cells [1]. They observed preservation of matured neuronal cells as well as boosted locomotor function when they grafted the iPSC-NPCs transduced with HSVtk into a rodent SCI model [1]. Thus, it indicated that only the tumorigenic cells were ablated after GCV injection [1]. The HSVtk/GCV system was adopted in clinical trials without any safety problems [164, 165].

Several studies demonstrated the effectiveness of iPSC-NPC grafting in chronic SCI notwithstanding the associated complications [1, 166]. Okano et al. elucidated the potential beneficial effect of iPS-derived NS/PCs for the repair of SCI and observed that careful preassessment of each iPSC clone prior to any clinical trial of human CNS illnesses was essential [167]. Uezono et al. described the efficacy of pretreatment against neural inflammation linked with iPSC-NPC grafting [168]. They observed that after iPSC-NPCs were grafted in this reformed milieu of SCI, the cells triggered copious synaptic connections with host neurons, which stimulated functional locomotor recovery [168].

## 9. Umbilical Cord Blood Cells

Umbilical cord blood cells (UCBCs) (Figure 1) have demonstrated to have therapeutic potential in various areas of medicine [169]. UCBCs are relatively easy to collect and possess distinctive features which make these cells exceptionally well customized for use as cellular treatments. UCBCs have a high rate of hematopoietic stem and progenitor cells; they are native of the immune cells and also possess nonhematopoietic cells that have therapeutic potentials [169]. Initial study demonstrated that human cord blood leukocytes were advantageous in reversing the behavioral effects of SCI, even when transplanted five days after injury [170, 171].

Also, human UCBCs transplanted into injured rat spinal cord models revealed that the UCBCs appeared in injured areas, but not in noninjured areas of rat spinal cords [170, 171]. Furthermore, the cells were never detected in analogous areas of the spinal cord of noninjured animals. These findings were coherent with the postulation that UCBCs migrate to and partake in the healing of neurological defects after SCI [170, 171]. Studies further demonstrated that transplanted human UCBCs differentiated into several neural cells, stimulated renewal of spinal cord tissue, and enhanced motor function in SCI rat models [172–175].

Moreover, molecular and ultrastructural analyses demonstrated that human UCBCs were capable of augmenting neuronal and oligodendrocyte survival in the injured zones [175, 176]. Cho et al. observed recovery of somatosensory evoked potentials as well as phenotypic differentiation of transplanted human UCBCS into oligodendrocytes [177]. Enormous quantities of non-ESCs are available in UCB comprising of a mixture of distinctive types of stem/progenitor cells, hematopoietic cells such as HSCs and CD34^+^/CD45^−^, and endothelial cells like CD133^+^ stem cells (Table 1) [3].

## 10. Methods of Transplantation of Stem Cells in SCI

Currently, local and intravascular approaches are the main methods of administering cellular therapeutics to the spinal cord [178]. Local administration is often attained via either direct cellular injection into the spinal cord parenchyma or intrathecal administration into the subarachnoid space [178]. However, intravascular approaches often include both intra-arterial as well as intravenous routes [178]. The intraparenchymal route demonstrated the greatest transplantation efficiency with several differentiated cells within the injured parenchyma [178]. Nevertheless, with the intrathecal route, few transplanted cells were detected on the surface of the lesion site as well as on other areas of the uninjured spinal cord and the transplanted cells differentiated into neurons, astrocytes, and oligodendrocytes [178].

In both routes above, cells which were transplanted cells were not detected at off-target sites or outside of the spinal cord [178]. In most studies that the transplanted cells were administered intravenously, cells did not migrate to the injury site in the spinal cord but rather migrated to the lung, spleen, and kidney [178]. Also, many mice in the intravenous group died soon after transplantation as a result of possible pulmonary embolism [178]. Furthermore, the study observed a similar pattern of graft survival, with signal loss or cell death happening in the first week after transplantation on longitudinal bioluminescence imaging [178].

Moreover, in a mouse contusive SCI model, neural stem/progenitor cell transplantation was compared between intralesional and intrathecal and intravenous administrations [109, 179]. Transplanted cells were highest in the intralesional hand-held injection group, and after approximately six weeks posttransplantation, cell luminescence declines to about 10% of their original level at the site of injury [109, 179]. In the intrathecal group, transplanted cell luminescence was scattered all over the subarachnoid space soon after transplantation [109, 180]. It was detected at the injury site pial surface one week later, and by six weeks, it had decreased to about 0.3% of the initial level [109, 180]. In the intravenous group, no grafted luminescent cells were found at the injury site but all of these mice exhibited cell accumulation in the chest, signifying pulmonary embolism [180].

## 11. Factors Influencing Spinal Injection Techniques and Challenges

Understanding the spinal cord structure as well as tissue properties, motion, blood supply, injury responses, and the properties of injection devices may assist in understanding the problems associated with therapeutic injections in SCI [14]. It is challenging to understand the importance of volume in spinal tissue. Nevertheless, a single nanoliter (*μ*l) occupies a sphere with a radius of 124 *μ*m, which is a significant space within spinal cord tissue [181]. A study revealed that the absolute volume of the human T8 spinal cord segment is 690 mm^3^ or 690 *μ*l [14, 181]. Some authors have demonstrated that the lesion volume in the spinal cord will be about 350 *μ*l using a theoretical dimensions of 3.5 mm radius × 18 mm length as a model cylinder of the T8 spinal cord segment and permit of 1 mm of the conserved tissue rim [181]. The same idea above can be applied to other segments of the spinal cord during injection of stem cells.

Fluid clearance is a possible means of augmenting compliance during spinal injections [14, 182]. A study in the brain revealed that some fluid clearance may occur via bulk fluid flow as well as diffusion along the extracellular spaces and absorption to the CSF or blood particularly via the white matter [14, 182]. It is proven that the extracellular spaces are open increasing this flow during focal edema in the CNS. Nevertheless, these extracellular channels comprising of membrane interstices as well as ground substance may be dilated after trauma resulting in acutely generated extreme pressures [14]. The rates of brain fluid clearance have usually been measured in hours and not minutes [14]. A study demonstrated that with very slow pressure injections of solutes, a diffusion rate of 44 *μ*l/5 min correlated with exceptional tissue conservation [14, 183]. Thus, at the above rate, it would take 114 min to administer 1 *μ*l [14].

Studies have shown that the fundamental forces generated within the spinal cord tissue during injection correlate with tissue's properties like compliance, elastic modulus, stress, and strain, as well as susceptibility to radial tensile stress—tearing stress—as a result of pressure gradients [14, 184, 185]. The injection of stem cells in animal spinal cord models usually involves the immobilization with frames, micromanipulators, syringes, and syringe pumps like those manufactured by Hamilton, Kopf, Stoelting, Harvard, and other companies [14].

On the other hand, human injection of stem cells involves medically approved devices, specifically those which are disposable [14]. This makes it more difficult to use innovative solutions during experimental injections. Thus, for human injections, factors such as practicability of usage in the operating room, quick assembly, reliability, potential for sterilization, reproducibility, nonobstruction of the visual field by the surgical microscope, and resistance to accidental perturbation or disarticulation once deployed within the spinal cord are often considered [14]. They are two types of injection techniques described in human studies [14].

The first ones are the hand-held syringe injection techniques, which are founded on the interoperator changeability based on the depth of placement, motion during injection, and injection rates. These techniques are simple, more rapid to deploy, and more flexible based on the approach angles [14, 186]. The second type is the use of a surgical operating table-mounted stereotaxic device. In this technique, enormous quantities of small injections were delivered into the damaged cord as well as the normal cord above and below the injured site [14, 187]. This device-stabilized technique of administering cells offers the capacity to accurately target single or multiple sites within the spinal cord just like in cranial stereotactic techniques [14, 187].

Preclinical grafting of cell in rodent studies has essentially utilized the stereotactic frame modified as a syringe and needle holder or free-hand injections [4, 180, 188]. The hand-held syringe injection technique often permits the surgeon to compensate for negligeable systolic and/or respiratory movements [109]. Clinically, syringe positioning device injections in subacute to chronic SCI have also been investigated [109]. Table-mounted syringe positioning devices or patient-anchored, retractor-based, syringe positioning devices which are often rigid or attached to a floating cannula are the two options for syringe stabilization [109]. The quantity of tissue dissection needed to anchor the device which could result in spinal instability like kyphosis is the possible shortcoming of the retractor-mounted or patient-anchored devices [109].

Several clinical trials utilized hand-held cellular injections after acute, subacute, and chronic SCI [109, 189]. The cell types that were used in these clinical trials included bone marrow MSC, activated macrophages, and autologous OECs [189–192]. In another study, the C4-T6 segment of the spinal cord was trimmed of scar tissues subsequent to the transplantation of OECs in the defect [189]. These authors observed improvement in motor function in the transplanted subjects [189]. Furthermore, long-term follow-up in at least one subject revealed the occurrence of a mass comprising of mucoid cysts with respiratory epithelium origin eight years posttransplant [191, 193]. In other studies, some cell types subsequently displayed substantial migration while others do not. SCs or bone marrow MSC displayed the ability to extemporaneously form linear bundles parallel to white matter tracts [14, 129].

## 12. Cell Transplantation and Spinal Cord Repair

As soon as SCI occurs, astrocytes proliferate as well as consolidate around the edges of the injury site to separate the damaged area from the surrounding healthy tissue [7]. Several studies have demonstrated that in the subacute phase, usually from one to two weeks after injury, reactive astrocytes migrate to the epicenter of the injury site and subdue inflammatory cells resulting in tissue repair as well as functional improvement [194–196]. Studies have further demonstrated that the extended reactive astrocytes around the injured perimeter triggers a fibroblast-like pericyte resulting in the formation of the astrocytic scar, the key inhibitor of CNS axonal regeneration during the later phase of the SCI [197–199].

Contrarily, some studies have demonstrated that the glial scar was obligatory to inhibit the spread of injury as well as essentially boost CNS repair [200, 201]. Fibrotic scarring was initially described to originate from meningeal cells after CNS injury. Nevertheless, studies demonstrated that PDGFR*β*-positive pericytes as well as CD13-positive endothelial cells are active sources of the cellular configuration of the fibrotic scar in SCI [202, 203]. Another study revealed that microvascular endothelial cells engulfed myelin debris via autophagy-lysosome pathway-triggered inflammation, angiogenesis, and fibrotic scar formation [204, 205].

After successful transplantation, implanted neural stem/progenitor cells (NSPCs) differentiate into neural cells that replace injured cells as well as offer local neurotrophic factors that aid neuroprotection, immunomodulation, axonal sprouting, axonal regeneration, and remyelination. Studies have shown that transplantation of NSPCs in the acute or subacute phases of SCI differentiate into oligodendrocytes, augmented the quantity of myelinated axons at the injure site, and boosted functional recovery [206–208]. Furthermore, studies showed that NSPC-derived myelin was fundamental in the remyelination process after SCI [208, 209]. Thus, this exhibits the significant role of remyelination in functional recovery triggered by stem cell transplantation approaches during SCI [209–211].

Lu et al. demonstrated that stem cells transplanted into the cavity and/or surrounding tissue regenerated as well as expressed neurotropic factors that triggered the growth of axons, both endogenous and graft-derived, across the lesion to form synapses as well as repaired spinal cord connectivity [212]. Studies have also shown that one of the mechanisms via which functional recovery happened in subjects with SCI was via neural plasticity or the capability of the CNS to regenerate its circuits over time [213–215]. Bonner et al. established that the neurons that differentiated from transplanted NSPCs prolonged axons as well as form new synapses with host neurons. They further indicated that the regenerated connections were mostly not exact reconnections of the lost neural circuits, but rather de novo circuits [216].

Assinck et al. demonstrated that oligodendrocyte precursor cells (OPCs) are primarily quiescent in the healthy CNS [217]. They indicated that OPCs are capable of proliferating and differentiating into mature oligodendrocytes in response to injury, which aid in remyelination [217] . Studies further showed that transplanted OPCs do not only complement the inadequate remyelination process of endogenous OPCs but also express neurotrophic factors that inhibited inflammation as well as promote axonal regeneration [218–220]. Studies have also proven that SCs myelinate peripheral nerve fibers and are capable of migrating into the injured spinal cord as well as boost remyelination after SCI [81, 188].

Several studies have demonstrated that transplanted SCs are capable of remyelinate axons as well as boost neural conduction just like OPCs [221, 222]. SCs are also capable of expressing growth factors, extracellular components, and adhesion molecules that triggered functional recovery after SCI [223–225]. Xu et al. demonstrated that NSCs transplanted into the lumbar ventral horn migrated to the central canal as well as triggered proliferation of ependymal cells and differentiated into neural precursors and neurons [226]. Salewski et al. established that ESC-derived NSPCs were capable of treating subjects with SCI without tumor formation [227].

**Figure 2:**
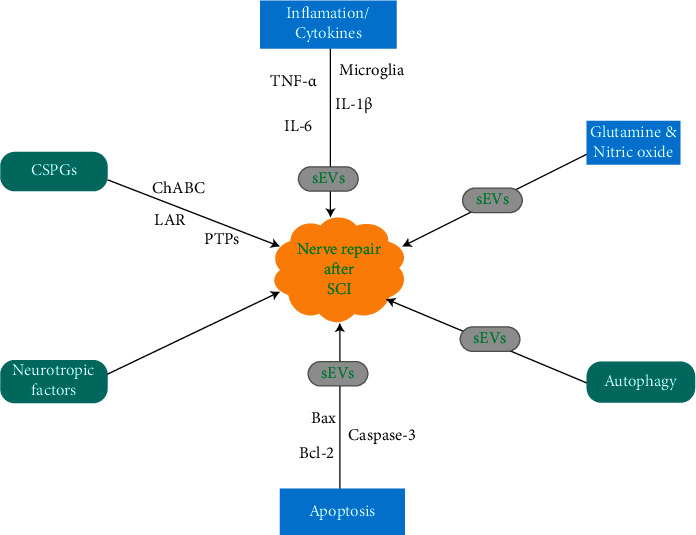
An illustration showing the nerve repair mechanisms after SCI. Nerve's lacking of self-repair. CSPGs: chondroitin sulfate proteoglycans; ChABC: chondroitinase ABC; LAR: leukocyte common antigen related; PTP*σ*: protein tyrosine phosphatase *σ*; sEVs: small extracellular vesicles.

Several studies have demonstrated that transplanted exogenous NSCs are capable of triggering neurogenesis in the spinal cord ependymal niche as well as boost the survival of the newly generated host neurons, which was analogous to the neurogenesis triggered in the brain SVZ via NSPC and MSC transplants [228, 229]. The key challenges associated with nerve repair are the lack of self-repair as well as neurotrophic factors, primary and secondary neuronal apoptosis, and factors that prevent the regeneration of axons locally (Figure 2) [230–232]. It was established that neurons that survive the initial traumatic damage may be lost due to pathogenic activities like neuroinflammation and apoptosis [232, 233].

Rong et al. demonstrated that NSC-derived small extracellular vesicles (sEVs) were capable of inhibiting neuronal apoptosis, microglia stimulation, and neuroinflammation resulting in the stimulation of functional recovery in SCI model rats [233]. They stressed that these outcomes above transpire as a result of neuronal autophagy [233]. SEVs are small vesicles expressed by cells that partake in cell-cell signaling via the transmitting of RNA, proteins, and bioactive lipids [234–236]. Studies have shown that the source of these vesicle correlated well with the expression of specific surface antigens [235–237]. Furthermore, several studies have demonstrated that sEVs generated by NSCs had therapeutic efficiency against ischemic, inflammatory, and neurodegenerative diseases [238, 239].

NSC-sEV was capable of inhibiting glutamate excitotoxicity *in vitro* as well as secondary SCI *in vivo* during pretreatment experiments (Figure 2) [233]. Also, NSC-sEV was capable of inhibiting neuroinflammation processes like microglial activation, nitric oxide (NO) expression, and cytokine production thereby stimulating autophagy (Figure 2) [233]. Thus, the antiapoptotic and anti-inflammatory actions of NSC-sEVs were directly dependent on the stimulation of autophagy (Figure 2) [233]. Several studies have demonstrated that although the pathogenic mechanisms of SCI are complex, inflammation and apoptosis are the two key processes that occur at the secondary phase of the injury [240, 241]. Several studies have demonstrated that after SCI, proapoptotic proteins Bax and cleaved caspase-3 are often elevated, while antiapoptotic Bcl-2 is normally decreased (Figure 2) [242, 243].

Furthermore, inflammation (Figure 2) involves triggering of microglia as well as elevation of neuroinflammatory cytokines like TNF-*α*, IL-1*β*, and IL-6 after traumatic SCI [240, 244].

Rong et al. demonstrated that LPS-induced NO generation by isolated microglia was downregulated by preincubation with NSC-sEVs [233]. Also, the secretory levels of proinflammatory cytokines were appreciably inhibited by NSC-sEV (Figure 2) pretreatment [233]. Furthermore, the quantity of activated CD68-positive microglia (Figure 2) was appreciably reduced in SCI pretreated with NSC-sEVs compared to the untreated injured spinal cord, signifying that NSC-sEV was capable of downregulating neuroinflammation *in vivo* as well as *in vitro [*233*]*.

Bradbury et al. demonstrated that mortification of chondroitin sulfate proteoglycans (CSPGs) by chondroitinase ABC (ChABC) was capable of breaking down the inhibitive barrier as well as stimulated endogenous pathological repair resulting in synapse reorganization as well as functional recovery in SCI subjects (Figure 2) [245]. Studies have demonstrated that a combination of stem cells and ChABC promoted functional recovery even in the chronic phase of SCI [246–248]. CSPG inhibition was facilitated by two members of the leukocyte common antigen-related (LAR) phosphatase subfamily and protein tyrosine phosphatase *σ* (PTP*σ*) [246–248]. Also, the LAR and PTP*σ* receptors were facilitated via the stimulation of oligodendrocyte differentiation and apoptosis by CSPGs in SCI [247, 249, 250].

## 13. Conclusions

Implanted stem cells are capable of differentiating into neural cells that replace injured cells as well as offer local neurotrophic factors that aid neuroprotection, immunomodulation, axonal sprouting, axonal regeneration, and remyelination. At the microenvironment of SCI, stem cells are capable of producing growth factors like BDNF and NGF which trigger neuronal survival as well as axonal regrowth. Although stem cells have proven to be of therapeutic value in SCI, the major disadvantage of some of the cell types is the risk for tumorigenicity due to the contamination of undifferentiated cells prior to transplantation. Local administration of stem cells via either direct cellular injection into the spinal cord parenchyma or via intrathecal administration into the subarachnoid space is currently the best transplantation modality for stem cells during SCI.

## Figures and Tables

**Table 1:** The immune players influenced by the various stem cell types at the injury microenvironment after transplantation in SCI.

Type of cells	Immune players influenced at injury milieu	Effects on recovery	Citations
*Neural stem cells (NSCs)*	FGF2 HGF CD133^+^/CD34^−^/CD45^−^ Neurogenin-2 GSI	Facilitatory Facilitatory Facilitatory Facilitatory Tumor inhibition	[55] [61] [62] [67] [72]

*Schwann cells (SCs)*	p75 GFAP S100 L1 N-CAM NGF BDNF GDNF CNTF IL-10 Fibronectin Laminin	Facilitatory Facilitatory Facilitatory Facilitatory Facilitatory Facilitatory Facilitatory Facilitatory Facilitatory Facilitatory Facilitatory Facilitatory	[3, 74] [3, 74] [3, 74] [3, 74] [3, 74] [78–81] [78–81] [78–81] [78–81] [86–88] [74] [74]

*Olfactory ensheathing cells (OECs)*	p75 GFAP S100 L1 N-CAM Fibronectin Laminin	Facilitatory Facilitatory Facilitatory Facilitatory Facilitatory Facilitatory Facilitatory	[3, 74] [3, 74] [3, 74] [3, 74] [3, 74] [74] [74]

*Ependymal cells*	EGF FGF2	Facilitatory Facilitatory	[107] [107]

*Human central nervous system stem cell (HuCNS-SC)*	Further studies needed	Facilitatory	[109]

*Erythropoietin-releasing neural precursors cells (Er-NPCs)*	Macrophages rhEPO 5-TH GAP43 BDNF NGF *β*-Tubulin III MAP-2	Inhibitory Facilitatory Facilitatory Facilitatory Facilitatory Facilitatory Facilitatory Facilitatory	[31, 112] [117] [31, 116, 117] [31, 118–120] [116, 122, 123] [116, 122, 123] [31] [31]

*Mesenchymal stem cells (MSCs)*	5-HT GAP43 BDNF VEGF FGF2 MAP-2 GABA-A BNP TGF-*β*	Facilitatory Facilitatory Facilitatory Facilitatory Facilitatory Facilitatory Facilitatory Facilitatory Facilitatory	[137] [128, 147] [4, 148] [4, 148] [4, 148] [4, 151] [4, 151] [4, 152, 153] [4, 161, 162]

*Induced pluripotent stem cells (iPSCs)*	GSI HSVtk GCV	Tumor inhibition Tumor inhibition Tumor inhibition	[1] [1] [1]

*Umbilical cord blood cells (UCBCs)*	CD34^+^/CD45^−^ CD133^+^	Facilitatory Facilitatory	[3] [3]

## Data Availability

No data was used for this paper.

## Abbreviations

GABA-A: *γ*-Aminobutyric acid
GSI: *γ*-Secretase inhibitor
BDNF: Brain-derived neurotrophic factor
BNP: Brain natriuretic peptide
CSF: Cerebrospinal fluid
CNTF: Ciliary neurotrophic factor
CNS: Central nervous system
CSPGs: Chondroitin sulfate proteoglycans
ChABC: Chondroitinase ABC
EGF: Epidermal growth factor
Er-NPCs: Erythropoietin-releasing adult neural precursors cells
ESCs: Embryonic stem cells
FGF2: Fibroblast growth factor-2
GCV: Ganciclovir
GDNF: Glial cell-derived neurotrophic factor
GAP43: Growth-associated protein-43
HuCNS-SC: Human central nervous system stem cell
HSVtk: Herpes simplex virus type I thymidine kinase
iPSCs: Induced pluripotent stem cells
LAR: Leukocyte common antigen related
MSCs: Mesenchymal stem cells
NSCs: Neural stem cells
NGF: Nerve growth factor
NSPCs: Neural stem/progenitor cells
NO: Nitroxide
OECs: Olfactory ensheathing cells
OPCs: Oligodendrocyte precursor cells
PNS: Peripheral nervous system
PTP*σ*: Protein tyrosine phosphatase *σ*
ROS: Reactive oxygen species
SCI: Spinal cord injury
SCs: Schwann cells
SVZ: Subventricular zone
sEVs: Small extracellular vesicles
TGF-*β*: Transforming growth factor-*β*
UCBCs: Umbilical cord blood cells
VEGF: Vascular endothelial growth factor.
